# The Role of Electronic Health (eHealth) Literacy in Patient Activation: A Sequential Block Regression Analysis Controlling for Sociodemographic Factors

**DOI:** 10.7759/cureus.97646

**Published:** 2025-11-24

**Authors:** Leonardo Bonifanti, Curtis Ko, Raymond L Ownby

**Affiliations:** 1 Psychiatry and Behavioral Sciences, Dr. Kiran C. Patel College of Osteopathic Medicine, Nova Southeastern University, Fort Lauderdale, USA

**Keywords:** chronic disease care, ehealth literacy, electronic health, low socio-economic strata, patient activation measure (pam-13)

## Abstract

Patient activation is defined by the knowledge, skills, and confidence to manage one’s health; it has been linked to better health outcomes and lower healthcare utilization. Despite its well-established benefits, the factors influencing patient activation are complex and not fully understood. One such factor, electronic health (eHealth) literacy, remains underexplored, particularly in underserved populations. eHealth literacy, which refers to the ability to access, understand, and apply health information from electronic sources, is increasingly vital in the modern healthcare environment. This study investigates the link between eHealth literacy and patient activation in adults with chronic diseases and lower socioeconomic status (SES). Data were drawn from 334 participants in a larger randomized controlled trial evaluating a mobile app for chronic disease self-management. eHealth literacy was measured using the eHealth Literacy Scale, while patient activation was assessed with the Patient Activation Measure (PAM), ten-item version (PAM-10). Sequential block regression analyses showed that eHealth literacy was significantly related to patient activation, after controlling for age, gender, and race. This has important implications for the design and delivery of healthcare interventions, suggesting that targeted efforts to improve digital health literacy could help bridge the activation gap, especially in socioeconomically disadvantaged groups. As healthcare increasingly shifts toward digital platforms, enhancing eHealth literacy could play a critical role in reducing health disparities and improving clinical outcomes in vulnerable groups.

## Introduction

In a healthcare landscape burdened with chronic conditions that require patients to actively participate in preventative and restorative measures for their care, patient activation is crucial for improving their clinical outcomes. Patient activation refers to the knowledge, skills, and confidence a patient has in managing their health [1]. Patient activation can be quantified using the Patient Activation Measure-13 (PAM-13) questionnaire, created by Judith Hibbard, and has been shown to be clinically reliable and valid [2]. Higher PAM scores correlate with several significant positive clinical outcomes, such as improved control of blood sugar as measured by HbA1c (hemoglobin A1c (glycated hemoglobin)), improvements in serum lipids as measured by high-density lipoprotein (HDL (high density lipoprotein) and triglycerides), as well as better health behaviors such as adhering to preventive screenings and healthier lifestyle choices [3-5]. Furthermore, patients with higher PAM scores tend to have fewer hospital admissions, readmissions, and emergency room visits, all of which reduce unnecessary healthcare utilization [6,7]. For example, patients with lower PAM scores are 1.4 times more likely to be hospitalized and 1.3 times more likely to visit the emergency room compared to those with higher values [8].

PAM is influenced by various intrinsic and extrinsic factors, but in a societal and medical arena that is increasingly digital, the role that electronic health (eHealth) literacy has in a patient's activation has not been fully clarified. eHealth literacy (eHeals) is defined as the ability to seek, find, understand, and appraise health information from electronic sources and apply that knowledge to managing or resolving health issues. In an ever-evolving technological landscape, it is increasingly important for patients to be comfortable with navigating, accessing, and synthesizing information found both online and in their electronic medical portals. Furthermore, telehealth has drastically increased in popularity following the COVID-19 pandemic, causing a lasting shift toward telehealth as a routine component of patient care [9]. As technology use rises, certain groups are disproportionately affected by a lack of access to it; among these are individuals with lower socioeconomic status (SES) [10]. Prioritizing individuals with lower SES is critical because this population often faces structural barriers to digital access, including limited internet connectivity, lower educational attainment, and reduced exposure to online health tools, all of which can constrain eHealth literacy. Since patient activation depends on an individual’s ability to understand and act on health information, examining eHeals within this group offers a more sensitive test of whether improving digital health skills could mitigate disparities in engagement, self-management, and health outcomes. We hypothesized that these individuals’ eHeals scores would have a significant impact on their feelings of engagement and being in charge of their healthcare. Although eHealth literacy has been tied to increased scores on some items related to the PAM, this study aimed to delineate the effects that eHealth has on PAM as a whole in patients with low SES, while controlling for demographic factors such as age, gender, and race [11]. The relationship between these two variables has not been thoroughly studied, especially in this subgroup of the population.

## Materials and methods

Study design and participants

This present study employed a cross-sectional survey design to examine the relationship between eHealth literacy and patient activation among adults with chronic health conditions. Data were drawn from participants enrolled in a larger randomized controlled trial designed to evaluate a mobile app for chronic disease self-management [12]. Participants (N=334) were recruited in both Fort Lauderdale, FL, and Atlanta, GA, from previous studies, clinics, and by word of mouth. At the Atlanta site, a paid recruiter visited local churches to recruit and screen potential participants.

Inclusion criteria required participants to be 40 years of age or older, currently receiving treatment for at least one chronic condition (e.g., cardiovascular disease, diabetes, and arthritis), and taking at least one prescribed medication. Participants also had to demonstrate health literacy below the 8th-grade level, as assessed by a short form of the Rapid Estimate of Adult Literacy in Medicine (REALM). Exclusion criteria included inability to provide informed consent or cognitive/psychiatric impairments that could interfere with participation.

The study protocol was approved by the Institutional Review Boards of Nova Southeastern University and Emory University, and all procedures adhered to the ethical standards outlined in the Declaration of Helsinki. Participants provided oral consent for screening procedures and written informed consent prior to participation in other study activities.

Measures

Electronic Health (eHealth​​​​​​​) Literacy

eHealth literacy was measured using the eHealth Literacy Scale [13]. These scales were administered via a computer-delivered interview, with all content read aloud to participants to reduce the possible impact of literacy level on their responses. Responses were scored on a multiple-choice self-report format, with higher scores reflecting greater eHealth literacy.

Patient Activation Measure

Patient activation was assessed using the 10-item Patient Activation Measure (PAM-10), a validated instrument evaluating individuals’ knowledge, skills, and confidence in managing their health and healthcare [1]. PAM scores were converted to a standardized metric ranging from 0 to 100, with higher scores indicating greater activation. Internal consistency in this study was acceptable (Cronbach’s α=0.87).

Demographic Variables (Covariates)

Participants self-reported their age (in years), gender (coded as 0=male, 1=female), and race/ethnicity (categorized as non-Hispanic White, Black or African-American, Hispanic, or Other). These covariates were included in regression models to control for demographic effects on patient activation.

Procedure

Participants completed the study survey as part of a comprehensive psychosocial and health literacy assessment battery embedded within the broader trial protocol. Surveys were administered either electronically via touchscreen computers or in person with research staff, depending on participant preference and literacy needs.

Data analysis

Data was analyzed using IBM SPSS Statistics version 29 (IBM Corp., Armonk, NY). Descriptive statistics were computed for all variables. Sequential block linear regressions were used to assess whether eHealth literacy significantly predicted patient activation after adjusting for demographic variables. In block 1, age, gender, and race were entered as covariates with the PAM-10 as the dependent variable. In block 2, eHealth literacy was added to assess its impact on patient activation.

To assess whether the assumptions underlying multiple regression were met, a series of diagnostic tests were conducted. Multicollinearity was evaluated using variance inflation factors (VIFs). Independence of residuals was examined using the Durbin-Watson statistic. Normality of residuals was assessed with a normal probability (P-P) plot of standardized residuals. Linearity and homoscedasticity were examined by inspecting a scatterplot of standardized residuals versus standardized predicted values. Finally, potential outliers were identified as cases with standardized residuals greater than ±3. The regression analysis was rerun with these cases removed. These diagnostic analyses confirmed that the data met the assumptions required for multiple regression analysis.

Psychometric properties of the eHealth Literacy Scale were evaluated by calculating the coefficient alpha for the scale in our sample. Model fit was assessed by examining R² and ΔR² values, and the significance of individual predictors was determined using t-tests and their related probabilities (p<0.05). Multicollinearity was evaluated using tolerance and VIF values, with thresholds of VIF <5 and tolerance >0.10 indicating acceptable levels. Assumptions of linearity, normality, and homoscedasticity were assessed by inspecting residual plots and distributions of standardized residuals.

## Results

The analysis aimed to assess the effect of eHeals on the PAM while controlling for potential confounding effects from age, gender, and race. A multiple regression analysis was conducted, with the PAM as the dependent variable and age, gender, race, and eHealth literacy as the independent variables. Coefficient alpha for the eHeals was 0.97, suggesting that eHeals had substantial internal reliability.

Regression diagnostics 

**Figure 1:**
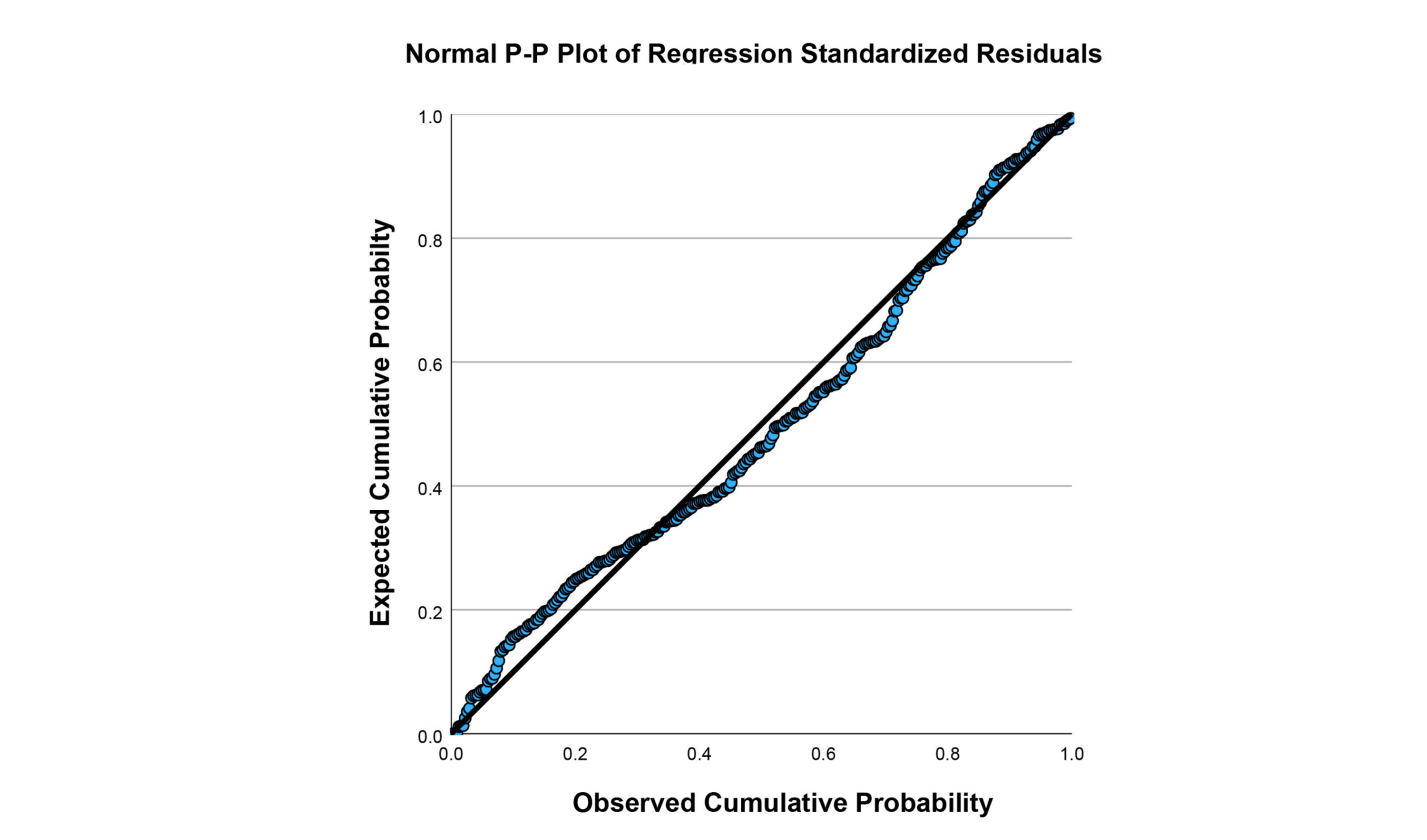
Normal P-P plot of regression standardized residuals P-P: probability-probability

**Figure 2:**
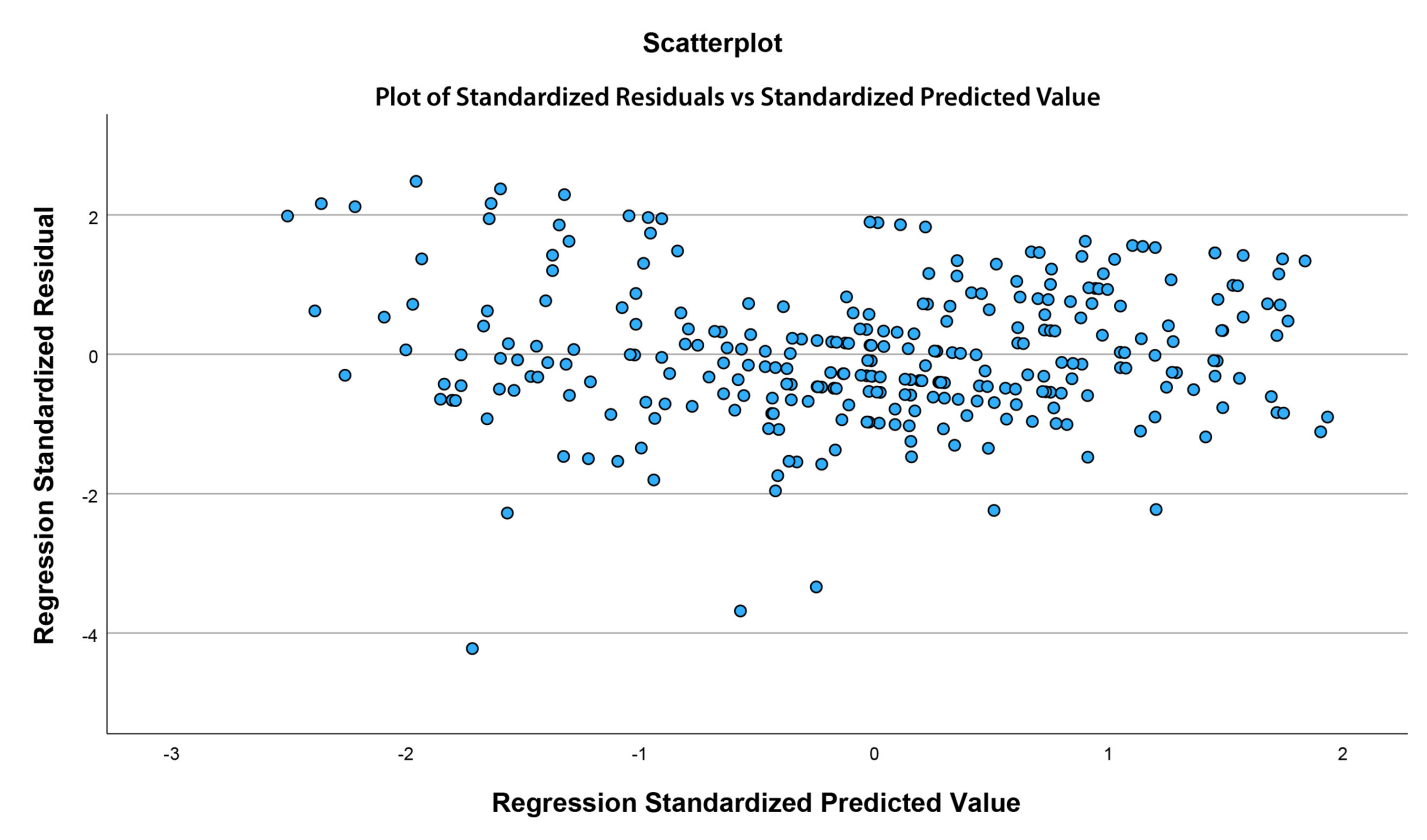
Scatterplot of standardized residuals versus standardized predicted values

Assumptions of multiple regression were examined to ensure the validity of the model. Multicollinearity was not a concern, as VIF values for all predictors were below 1.10 (maximum=1.06). The Durbin-Watson statistic was 1.97, indicating that residuals were independent and showing no evidence of autocorrelation. Examination of the normal probability (P-P) plot of standardized residuals (Figure 1) showed that points closely followed the diagonal line, suggesting that residuals were approximately normally distributed. A scatterplot of standardized residuals versus standardized predicted values (Figure 2) showed a random distribution of points with no systematic pattern or funnel shape, indicating that the assumptions of linearity and homoscedasticity were met. Together, these results support that the assumptions of normality, independence, and absence of multicollinearity were adequately satisfied.

Review of residuals identified three cases with standardized residuals greater than three. Removal of this small number of potential outliers did not alter the pattern or significance of the results.

Model overview

The initial model, which included age, gender, and race as predictors, explained only a small portion of the variance in PAM scores (R²=0.02, F(3, 292)=1.88, p=0.13). This model was not statistically significant, indicating that demographic factors alone did not meaningfully predict activation. When eHeals was added in the second block, the model’s explanatory power significantly improved (R²=0.08, ΔR²=0.07, F(1, 291)=20.69, p<0.001). This finding suggests that eHealth literacy accounted for an additional 6.5% of the variance in activation beyond that explained by demographics alone.

Model fit and interpretation

The final model, which included eHealth literacy alongside age, gender, and race, showed that eHealth literacy was a significant predictor of patient activation. While the demographic-only model (Model 1) explained very little variance (R²=0.019) and was not significant, adding eHealth literacy (Model 2) substantially increased the model’s fit (R²=0.084, adjusted R²=0.071, p<0.001). These results indicate that individuals with higher eHealth literacy scores tend to report greater patient activation, even after controlling for demographic variables (Table 1).

**Table 1:** Model summary PAM: Patient Activation Measure

Model^c^	R	R^2^	Adjusted R^2^	Std. error of the estimate	Change statistics					Durbin-Watson
					R^2^ change	F change	df1	df2	Sig. F change	
1	0.138^a^	0.019	0.009	4.67350	0.019	1.879	3	292	0.133	
2	0.290^b^	0.084	0.071	4.52345	0.065	20.694	1	291	0.000	1.969
^a^Predictors: (constant), race recoded, age, and gender recoded										
^b^Predictors: (constant), race recoded, age, gender recoded, and eHeals.0: eHeals										
^c^Dependent variable: PAM.0: PAM (added)										

Table 2 details the contribution of each predictor within the model. These coefficients indicate that eHealth literacy emerged as the sole statistically significant factor, with higher eHealth literacy scores positively associated with greater patient activation, while age, gender, and race were not significant independent predictors.

**Table 2:** Coefficients for predictors of PAM PAM: Patient Activation Measure; VIF: variance inflation factor

Model^a^		Unstandardized coefficients		Standardized coefficients	t	Sig.	Collinearity statistics	
		B	Std. Error	Beta			Tolerance	VIF
1	(Constant)	25.549	2.495		10.238	0.000		
	Age	-0.061	0.032	-0.109	-1.870	0.062	0.991	1.009
	Gender recoded	0.730	0.552	0.078	1.324	0.187	0.972	1.029
	Race recoded	-0.933	0.813	-0.067	-1.147	0.252	0.975	1.025
2	(Constant)	19.703	2.736		7.202	0.000		
	Age	-0.033	0.032	-0.059	-1.024	0.307	0.954	1.048
	Gender recoded	0.747	0.534	0.080	1.400	0.163	0.972	1.029
	Race recoded	-0.758	0.788	-0.055	-0.962	0.337	0.973	1.028
	eHeals.0: eHeals	0.144	0.032	0.260	4.549	0.000	0.961	1.040
^a^Dependent variable: PAM.0: PAM (added)								

Finally, model comparison results, presented in Table 3, confirm that the addition of eHealth literacy significantly enhanced explanatory power relative to the baseline demographic model (F change=6.678, p<0.001).

**Table 3:** ANOVA results for model comparison PAM: Patient Activation Measure; ANOVA: analysis of variance

ANOVA^a^						
Model		Sum of squares	df	Mean square	F	Sig.
1	Regression	123.150	3	41.050	1.879	0.133^b^
	Residual	6377.755	292	21.842		
	Total	6500.905	295			
2	Regression	546.589	4	136.647	6.678	<0.001^c^
	Residual	5954.317	291	20.462		
	Total	6500.905	295			
^a^Dependent variable: PAM.0: PAM (added)						
^b^Predictors: (Constant), race recoded, age, and gender recoded						
^c^Predictors: (Constant), race recoded, age, gender recoded, eHeals.0: eHeals						

Taken together, these findings underscore the central role of eHealth literacy in promoting patient activation and suggest that interventions aimed at improving eHealth literacy might be effective in enhancing patient engagement.

## Discussion

This study explored the association between eHealth literacy and patient activation in a sample of adults managing chronic health conditions, with particular attention to individuals of lower SES and limited baseline health literacy. After adjusting for sociodemographic variables, we found that eHealth literacy was a significant and independent predictor of patient activation. In contrast, age, gender, and race were not significant contributors in the final regression model.

These findings suggest that individuals with stronger digital health literacy skills are more likely to feel knowledgeable, confident, and engaged in managing their own health, regardless of traditional demographic risk factors. This aligns with the growing body of literature indicating that digital competence is increasingly central to effective self-management in modern healthcare environments. Previous studies have shown that higher eHealth literacy is associated with improved communication with providers, better health behaviors, and more satisfaction with shared decision-making processes [10,11]. Our results extend this work by demonstrating that these digital competencies are meaningfully associated with overall patient activation, a validated marker of engagement and self-management capacity.

Interestingly, demographic variables such as age and race were not independently associated with patient activation after accounting for eHealth literacy. This suggests that interventions aiming to enhance eHealth literacy may serve as a powerful equalizer, potentially mitigating health disparities associated with demographic characteristics, themselves often linked to lower activation and poorer outcomes.

This study has important implications for public health and clinical practice. As the healthcare system continues to transition toward tele-health, online patient portals, and app-based interventions, patients must navigate an increasingly complex digital landscape. Enhancing eHealth literacy through structured interventions, especially in underserved populations, could significantly boost patient activation and, by extension, improve health outcomes. The original trial protocol from which our dataset was drawn is itself an example of such a targeted, tablet-based intervention to improve literacy and engagement [12]. Future implementation efforts should prioritize scalability and cultural tailoring to maximize reach and impact.

Nevertheless, several limitations to our study should be acknowledged. First, the cross-sectional design limits the ability to infer causality between eHealth literacy and patient activation. It is possible that individuals who are more activated may be more motivated to seek and utilize online health information, suggesting a potentially bidirectional relationship. Second, although we controlled for demographic variables, certain contextual factors, such as access to reliable internet, frequency of technology use, and prior experience with digital health tools, were not measured and may meaningfully influence the observed associations. Third, our sample was drawn from a trial targeting individuals with low health literacy, which may limit the generalizability of findings to broader populations. Future research should incorporate these contextual variables and employ longitudinal or mixed-methods designs to clarify the temporal and behavioral mechanisms linking eHealth literacy and activation.

Future research should explore these dynamics longitudinally and evaluate the effectiveness of eHealth literacy interventions in improving activation and clinical outcomes. Additionally, qualitative studies could provide insight into the lived experiences of digitally underserved patients and inform strategies to reduce digital divides in healthcare.

## Conclusions

This study underscores the pivotal role of eHealth literacy in shaping patient activation among adults managing chronic conditions, particularly within socioeconomically vulnerable populations. Even after controlling for age, gender, and race, digital health literacy remained a significant and independent predictor of activation. These findings suggest that improving patients' ability to access, understand, and use digital health information may be a critical and actionable pathway to enhance engagement, promote self-management, and ultimately improve health outcomes. As healthcare continues to evolve toward digital platforms, equitable access, and competency to use eHealth tools must be prioritized to ensure no patient is left behind.
